# Instinct and Reason; or, the Intellectual Difference between Man and Animals

**Published:** 1851-07-01

**Authors:** James Quilter Rumball


### INSTINCT AND REASON;

### OR,

### THE INTELLECTUAL DIFFERENCE BETWEEN MAN AND ANIMALS.

BY JAMES QUILTER RUMBALL, ESQ., M.R.C.S., L.A.C., ETC.

WHAT is mind? Of whom shall I inquire? Of the metaphysicians? What is metaphysics?

I have read that when Sylla conquered Athens, a box of manuscripts formed portion of his plunder. They were submitted for translation to one Titus Andronicus, of Rhodes, a learned linguist of that time. They proved to be the works of Aristotle. There were two bundles; the first contained his "physics." This being rendered into Latin, the second was examined, and translated also; but when finished, it was found impossible to give it a local habitation or a name. It seemed to treat of everything, but to realize nothing; glancing everywhere, resting nowhere; an air-drawn dagger, vanishing into thin air even while we clutch it: and so the good man, with rare modesty, gave a name to it which not only acknowledged his own entire ignorance of the subject, but prophesied a like blissful state to all who should hereafter enter on this trackless sea. He wrote on it "Ta meta Ta physica, *τα μετα τα φυσικα.*" *That which follows the physics!* And the labours of two thousand years have only served to confirm the aptness of the title; for, down even to our day, the science of mind is as little understood, and as much contested, as it was in the time of Andronicus of Rhodes.

It would be fruitless toil to register the chronological failure of all metaphysicians from Plato to Paley, who have attempted to fix their wandering thoughts to tell of others, or to know themselves; but it is absolutely necessary to a clear stage, that we seek from their own mouths, reasons enough and good, for one more voyage on the unknown sea—one more journey o'er this moral Sahara.

What is mind? Is it identical with soul? Is it even a first principle, breathed into man's nostrils with the breath of life, and manifested by his animal organism? or is it, as Lawrence writes, a mere consequence, the result of organization, the product of matter?

If I ask Chalmers, he tells me that I must not even investigate it as I would physical subjects, or even morals; for "mental science (he says) is as distinct from all other sciences, including the ethical and logical, as our notions of things are from the things themselves."*

He denies that we can gain any knowledge of our own minds by self-examination. In examining the feeling of anger, for instance, page 61, he says, "The moment that I turn my eye inwardly for that purpose, the thing that I am in quest of takes flight and disappears;" again, page 75, "All that is known, all that can be known of the mind, is the various states, whether of intellect or emotion, into which it passes, and to which states it is primarily brought, by converse, not with itself, but with objects apart from itself;" and, "According to this view, it is memory which supplies us with all the materials of mental philosophy." Is not the above full of contradictions? How am I to become acquainted with "the various states" of my mind, but by "turning my mental eye" upon them as they pass? How am I to remember unless I previously perceive?

Dr. Brown considers mind "as one substance capable of existing in a variety of states,"† which seems as perfect a contradiction in terms as could well be penned.

Dr. Reid understands by mind, "that in us which thinks, remembers, reasons, wills"—not a word about "feels." He declares that "Every man must have ideas, for he that doubts it thinks, and to think is to have ideas." "It is certain that Plato had his doctrine as well as the name *Idea* from the school of Pythagoras; we have still extant a tract of Timæus, the Locrian, a Pythagorean, concerning the *Soul of the World*, in which we find the substance of Plato's doctrine concerning ideas. "They were held to be eternal, uncreated, immutable forms or models, according to which the Deity made every species of things that exists, of an eternal matter." These philosophers held that there are three first principles of all things.

1st.—An eternal matter, of which all things were made.

2nd.—Eternal and immaterial forms, or ideas, according to which they were made.

3rd.—An efficient cause, the Deity who made them.

The philosophers of the Alexandrian school placed all these in the *mind of the Deity*; "the eternal models were theirs and not Plato's."‡ Aristotle taught that all the objects of our thought enter at first by the senses; but "the mind does not receive the object, only the image, as wax receives the impression, but not the seal." "When they become objects of memory or imagination, they get the name of phantasma."§ "His system was called the peripatetic." Democritus and Epicurus held that all bodies continually send forth slender fibres or spectres from their surface, of such extreme sublety, that they easily penetrate our gross bodies, or enter by the organs of sense, and stamp their image on the mind. The *sensible species* of Aristotle were forms without matter; the species of Epicurus were composed of a very subtle matter."||

* Sketches of Moral and Mental Philosophy.

† Lectures on the Philosophy of Mind, page 67.

‡ Essays on the Powers of the Human Mind, page 9.

§ Ibid. Curiously enough a phrenological chart was published at Venice, with cranial delineations, on which, where we now place Ideality, we find Phantasma marked.

|| Page 10.

Dr. Reid thinks that we *can* acquire a knowledge of our own minds by "an accurate reflection upon its operations," "and by paying due attention to the course of human actions and conduct. The actions of men are effects; their sentiments, their passions, and their affections, are the causes of those effects, and we may in many instances judge of the cause from the effect." Our social affections are instanced as thus demonstrable. He divides the mind into two classes, "understanding and will." Under the will, he comprehends our active powers and all that lead to action, as "appetites, passions, affections,"* evidently confounding wish with will. He "cannot understand how the mind perceives objects, and declares his conviction that our original faculties are all unaccountable."† He calls "Idea" the creature of philosophy, about which nothing can be known; thereby agreeing with Locke, and opposing Mallebranche, Descartes, and Bishop Berkeley, who all believed the existence of an external world to be incapable of proof, but consenting to the dogma of Descartes, "*cogito ergo sum*," "I think, therefore, I am," as the alpha and omega of all our knowledge. Berkeley disbelieved in the existence of matter. Hume denied the existence both of matter and mind."‡ In short, so absurd does Reid think the metaphysical speculations of all who have preceded him, that at page 111 he sums up thus:—

"Some philosophers will have our ideas or part of them to be innate, others will have them to be all adventitious; some derive them from the senses alone, others from sensation and reflection; some think they are fabricated by the mind itself, others that they are produced by external objects; others that they are the immediate operation of the Deity; others say that impressions are the causes of ideas, and that the causes of impressions are unknown; some think that we have ideas only of material objects, but none of minds, of their operations, or of the relations of things; others will have the immediate object of every thought to be an idea; some think we have abstract ideas, and that by this chiefly we are distinguished from the brutes; others maintain an abstract idea to be an absurdity, and that there can be no such thing. With some they are the immediate objects of thought, with others the only object." §

So much for Reid's opinion of other metaphysicians. But does he improve upon them? Hear what he says of the will. "A child cannot be said to *will* to suck, because it has not reasoned about the complex operation." Again, logical and profound as he is, the following passage, page 472, shows him to be as confused and mistaken as any who went before him: "When a man beats a drum or plays a tune, he has not time to direct every particular beat or stop by a voluntary determination, but the habit which may be acquired by exercise answers the purpose as well." Oh! Reid, Reid; matter is quicker than thought, is it? Muscles than mind? *How is it when a man plays a quick passage at sight?* proh pudor!

Mind, says Dugald Stewart, is "that which feels, which thinks, and which has the power of beginning motion." This is denied, of course, by the materialists, who attribute these properties or functions to the brain.

I have already stated that Hume denied the existence both of matter and mind. In Book I., Part I., Lect. 1, of his Treatise on Human Nature he undertakes to prove two positions:—

1st.—That all that is called human knowledge (meaning demonstrative knowledge) is only probability.

2nd.—That this probability when duly examined, evanishes by degrees,

* Essays on the Powers of the Human Mind, p. 32. † Ibid., p. 564.
‡ Ibid. p. 97. § Ibid. p. 409.

and leaves at last no evidence at all; so that, in the issue, there is no ground to believe any one proposition rather than its contrary; and that "*all those are certainly fools who reason or believe anything.*" And yet he himself is *fool* enough to reason on the subject, and expect us to believe him! Kant and others contend for a merely ideal world. Hobbes, though a materialist, admitted no knowledge of an external world. According to him all that we know is the "seeming," the "apparition," the "aspect," the "phenomenon," the "phantasm" within ourselves; and this subjective object of which we are conscious, and which is consciousness itself, is nothing more than the "agitation" of our internal organism, determined by the unknown motions which he supposes constitute the world without. He admits no knowledge of an external world.*

Bayle agrees with Hume that we have not arrived at certainty in any branch of knowledge. Hume says we cannot!

Locke teaches that we have no ideas which do not arise from impressions on the senses, or from reflections on our own thoughts and feelings.

Descartes seems to have been misunderstood. In his Letters, No. 99, he declares that "He never said or thought that the mind needs innate ideas, which are something different from its own faculty of thinking; but as he observed certain thoughts to be in his mind, which neither proceeded from outward objects, nor were determined by his will, but merely from his own faculty of thinking, he called them innate ideas, to distinguish them from such as are adventitious, compounded by our imagination; he calls them innate in the same sense in which *generosity is innate in some families, gout and stone in others.* Because the children of such families come into the world with a disposition to such virtues, or to such maladies."

Locke clearly proves that none of our ideas are innate, for neither idiots nor children have them.

According to Leibnitz, it matters not that all phenomena have been called dreams, "since experience shows that we are not disappointed in the measures which we take concerning phenomena, when these phenomena are founded on the principles of reason."

Cousins, whose philosophy excites considerable sensation at the present time, resolves all things into three principles:—

1st.—The one which is infinite—absolute cause—pure thought.

2nd.—The many which is finite—phenomena—relative cause.

3rd.—The combination of these two, which is intelligence.

God and mind are of the first. God, as he is cause, is able to create; as he is absolute cause, he must create.

"*We* create as often as we exert our free causality."

"In creating the universe, he does not draw it from nothing, he draws it from himself. To create is not to make something out of nothing, but to originate from self." †

Schelling argued that "there is a capacity of knowledge above consciousness, and higher than understanding." But Hogel, his disciple, confesses that "this pure or undetermined existence is synonymous with pure nothing."

Cabernis, a pupil and friend of Condillac, asserted the double action of the nerves, and considered sensation as a *faculty of the nerves.* Lawrence, therefore, follows him in asserting mind to be the "result of organization."

Boutkoe, La Peyronie, and Louis, place the mind in the corpus cal-

* Leviathan, Bauchy's Collection. Edinburgh Review, vol. iii. p. 331.

† Ibid. p. 313.

losum; Vieussens, in the centrum ovale; Digby, in the septum lucidem; Drellincourt, in the cerebellum, and Soemmering, in the fluid of the ventricles.

Reid says, "we have, or we believe we have, an immediate knowledge of materiality," and he proves it by this anology; "we are conscious of consciousness, therefore it exists! we are conscious of external existence, therefore it exists."

Against the above, place Descartes' celebrated ultimatum, "Cogito ergo sum—I think, therefore I am;"—follow this up by examining Dugald Stewart, who refers almost all our mental powers to attention; and declares that imagination is the result of habit, and no more given by nature than wit or fancy; and then examine Brown, who asserts that "attention to objects of sense appears to be nothing more than the co-existence of desire with the perception of the objects to which we are said to attend." "But there is no operation of any power distinct from the desire and perception themselves."* Compare the whole of the foregoing, which if not already, might be increased into, the most tedious and confused series of antagonisms ever committed to paper; and can we feel surprised that the late Charles Mathews, of facetious memory, defined metaphysics to be "a discourse about what we do not understand, addressed to those who do not understand us;" or that Dugald Stewart should have combated the doctrines of Reid; and that Brown, the disciple of both, should have pronounced "all that was claimed as original and most important in their philosophy to be nought but a series of misconceptions, only less wonderful in their commission than in the general acquiescence in their truth;" or that a still later writer in the "Edinburgh Review" (Sir William Hamilton, I believe,) should have characterized Dr. Brown's theories as containing "Radical inconsistencies in every branch of the subject," "endless mistakes," and "frequent misrepresentations"? Jam satis! We are compelled once more to revert to our first inquiry, What is mind? and failing its discovery among all preceding philosophers, let us turn to Dr. Gall, and see if he is able "Inter hos, tantas componere lites."

What is mind? What is matter? What is any ultimate? No man ever has informed us, or ever can. Beyond the properties of matter, the effects of time, or the results of mind, we know nothing. Of their hidden essences we shall know nothing till time merges into eternity, and the future becomes the past; until we see God face to face, and the veil of the temple shall be rent. It is with the properties of mind, therefore, that we have to deal; and in assuming mind to be immaterial, we are borne out by the fact that its results are so. The properties of matter are few and simple. It has form, size, and substance; it can be weighed and measured; it occupies space, and is impenetrable—that is, no one atom of matter can occupy the portion of space filled by another. But who can reduce into inches, yards, or feet, a mother's love or childhood's joy? Who can give me, in pounds or ounces, the weight of my fear, my fancy, or my grief? If, then, the very terms we use to describe objects of sense are manifestly ridiculous and absurd when applied to mental operations, we may safely assert that mind is not matter, but that it is something without form, size, or substance, and we employ the word "immaterial" to describe it.

The same argument applies to life. Who can believe that a grain of corn, consisting of charcoal, some gases, and some salts, planted in the soil, which is composed of like materials; shall not only commence an activity that matter never shows (for its very essence, as far as we know

* Brown's Lecture, p. 198.

it, is to be incapable of commencing motion, vis inertia being perhaps its most prominent attribute) who can comprehend how the small seed shall send out roots from itself into the dead earth; suck up by their means, and select that aliment—and that alone—which is suited to its nature, and convert water, earth, and air, into every variety of herb, tree, fruit, and flower, that covereth the earth with beauty—ever changing, ever new—flung up into the air, or through the soil, far away from the small centres whence they bud, living and growing long after those centres have died and disappeared—with which in its lifetime they had no connexion, and of which they retain no similitude? No! life is evidently a second agent; an immaterial principle in the hands of Deity, whereby he organizes the earth and garnishes it; lying quiescent in the ovum or the seed, until favouring circumstances of light, or heat, or moisture, give it birth; when it runs its course like a wound-up spring, and fades away and dies, when its allotted time arrives.

> "The smallest flower that blows
> Is past our finding out;
> Whence come, and where it goes,
> Can we resolve the doubt?"

But although mind must be an immaterial principle or nothing, it does not follow that it is synonymous with soul; or should it even be so, it is illogical to claim a soul for brutes, because we assert that they have minds. The tree lives, so does the elephant; and yet the principle of life in the one may be as different in its nature from the other, as are the elements and properties of their bodies. To allow to Deity variety in his material agencies, and deny them to his spiritual, would be unsound in argument and absurd in its results. That brutes think and feel, no one can deny; that thought and feeling can either be weighed or measured, no one will assert; they have therefore within them an immaterial principle similar to man's, but an analysis of its attributes will convince us that though similar it is not the same.

We shall simplify the inquiry, even if we do not shorten it, if we begin with the beginning, and trace up from the simplest forms of being the successive stages of development, until we arrive at the most complex of which we have any cognizance.

Philosophers have classified nature under three heads—the mineral, the vegetable, and the animal. The first exhibits all the properties above enumerated as belonging to matter. The second, or vegetable kingdom, possesses also these properties; that is, it has form, size, and substance; it can be weighed and measured. But it has more than these,—it has life! it lives by means of an internal organized apparatus, by whose action it takes up from surrounding earth and air, elementary unorganized matter, which it assimilates to its own nature, converts to its own purposes, and parts with when its race is run, to fall back into their original inertness, until some other living thing wants them, seeks, and finds them.

Whereas, the mineral, therefore, was created without life, so will it cease to be, without death; as God made it so would it remain for ever, unless affected by external forces, mechanical or chemical. The granite on the mountain, and the clay at its base, would always retain the same size, density, weight, and position, were nothing to infringe upon or change them. The very sea maintains its ever-flowing, never changing identity.

> "Time writes no wrinkle on thine azure brow,
> Such as creation's dawn beheld, thou rollest now."

And the inconstant air, raging or still, in summer breeze or storm, is still and ever composed of twenty-seven parts of oxygen and seventy-three parts nitrogen; whose weights and properties are, as they ever have been, unchangeable and unchanged. But the vegetable, although created as a class, and so far lasting from the world's first to its last; has a definite term assigned to its individual existence; and although the germ of every oak may have been in the original acorn, each tree commences its own independent career, in its own appointed time, and fills the spot and hour destined for it before time was.

The animal kingdom exhibits all the laws both of the mineral and the vegetable. It has form, size, and substance. It can be measured and weighed. It was created as a class, and will last whilst the world lasts. It lives as an individual and dies: it is nourished by means of an internal organized structure, which takes up and applies to its own use suitable nourishment from surrounding objects; but it also has its own specific properties, although philosophers have failed in finding them. By some it has been said that the animal differs from the vegetable in that it has gluten. But the mushroom and onion and others have gluten! Some say animals have stomachs, vegetables none: but the zoophytes have far less appearance of stomachs than the pitcher-plant or fly-catcher! Even locomotion belongs not to the lower class of the animal kingdom. What then distinguishes the animal as a class from the vegetable as a class? *Consciousness*, and this only. Pluck a leaflet from the sensitive plant, and the wildest casuist will not assert that the root thereof cognizes the wrong; but wound the remotest fibre of the dullest animal, and the whole frame sympathises. The plant is injured and knows it not. The animal feels, and knows it feels, however it may be wanting in power to express.

Consciousness, then, is the corner-stone of the mental temple. Upon it is laid, one by one, the varied faculties and feelings which build up the animal world until it reaches to its apex—man.

> "Distinguished link in being's endless chain,
> Midway from nothing to the Deity."—YOUNG.

Man! the microcosm of all around him, exhibits, we shall find, all the attributes of all living things; and beyond these a moral development that separates him from the noblest brute, assimilates him to the angels, and indicates his immortality.

It has been already argued that both man and brutes have an immaterial thinking principle, manifested by organic structures; that we know nothing of this principle except in its manifestation; and that it is essential to examine into the nature of the manifesting structures if we would understand these manifestations.

The very lowest species of animals have a nervous system; semifluid and half-organized it may be, but it is nevertheless the chaos, the "indigesta que moles" out of which the finer fibres and cerebral development of the higher animals are evolved. Vegetables exhibit no trace of this. Should it even be contended that I have failed to prove the immateriality of mind, or, except by analogy, the consciousness of animals, yet here, in the anatomical analysis, we shall find neither difficulty nor doubt. As nerve, so sensibility; as brain, so mind, will be found strictly and demonstratively true, throughout every link of the whole animal chain.

From the long abdominal nerve of the worm up to the complex system of man, we shall find progressive additions as the animal intelligence increases.

The zoophites have the nervous system in its simplest form, that is jelliform, and yet they exhibit consciousness and volition.

"Ehrenberg thought he discovered a series of small gray nervous masses in animalcule, which he considered ganglions."

In the ascaris* a single thread passes along the body, but no ganglia are discoverable as yet.

Earth-worms have a double nervous chord and a ganglion at each ring.

In the crustacea the brain is typified.† "In the caterpillar there are many ganglia," (*i. e.*, small nervous nodules, composed of white and gray matter, such as the human brain is composed of, whilst the gray or cineritious matter is never found in the nerves.) "In the chrysalis we find a notable shortening of the chain of ganglia." In the papilio brassica Herold found "the whole chain of ganglia scarcely half as long as in the caterpillar; the sixth and seventh had altogether disappeared. The second, third, fourth, and fifth were united so as to form only two ganglia, and the ganglion above the œsophagus was composed of two large lobes, each of which gave rise to a large optic nerve."‡

Thus, as the caterpillar progresses to the butterfly—from the lower to its higher stage of existence—it is found to gather nervous matter into its head to form a brain; to see, and to fly, and propagate. This is the type of the whole, and the child's development is equally instructive.

In the head of the fœtus during the first month after conception, the whole head, brain and all, is gelatinous and transparent.

In the rabbit it is so to the fourteenth day.

In the chicken to the end of the second day.

In the second month, or during the fifth and sixth week (in the human fœtus), vessels are seen on and in the brain, and a few limpid and transparent globules formed by the pia mater. In the seventh and eighth week the head and spine lose their transparency.§

The dura mater is visible; the brain is like the white of an egg. *The cerebellum still divisible into two.*|| The cerebral hemispheres commence; there are no nerves, no annular protuberances, commissures, corpus callosum, &c.

As the fœtus progresses, additions are made to the brain as well as to the body; the eyes appear, and so do the nerves which are to supply them. The senses of smell, and hearing, and taste, all undergo a progressive development, until the child is born, and even after that; for the brain of an infant is not half so large, nor so fibrous, nor so perfectly developed as that of a full grown man, and its mental development maintains a precisely similar ratio.¶ As the brain of man is thus proved to be built up by degrees, so does it decline. The brain of an old man not only measures less, but weighs less than it did in the prime of life. Mr. Magendie ascertained that in persons above the age of seventy, the specific gravity of the brain is less on an average by a fifteenth than in adults."**

In fishes the brain is composed of egg-shaped ganglia, perfectly distinct, and only united together by small nervous filaments. "From the brain uniformly proceed the nerves of hearing, sight, and smell; and, consequently, the number of its principal divisions is always fixed at three."††

In addition to this cumulating evidence, we find that "the brain of man is larger in proportion to the spinal marrow than it is in any animal;"‡‡ or, as Tiedeman has it, page 143, "L'homme est celui de tous les

* See Plate. † Tiedeman.
‡ Carus, Comparative Anatomy, vol. i. p. 89.
§ Tiedeman, p. 13. Anatomy of Fœtal Brain. || Ibid. p. 22.
¶ For the successive stages of development in the human brain, see Plate.
** Mayo, p. 251. †† Carus, § 290. ‡‡ Soemmering.

animaux qui a l'encephale le plus volumineuse en égard à la masse de sa moelle epinière." Whilst Richerand goes so far as to say, "Of all animals man has the most capacious skull in proportion to his face; and as the bulk of the brain is always of a size proportioned to that of the osseous case which contains it, the brain is also most bulky in man. This difference of size between the cranium and face may be taken as the measure of the human understanding, and of the instinct of the lower animals. The stupidity and ferocity of the latter are greater according as the proportion of these two parts of the skull vary from those of the human head." Richerand's Physics, 2nd edit. p. 322. And, although opinions differ respecting the absolute size of the various brains of animals and man, yet all agree that the brain of man is larger, when compared with his nervous system generally, than that of any other animal. All agree that whatever the mind may be, the brain and the brain only manifests it. That in proportion to the comparative size, complexity, and composition of the brain, is the amount, power, and variety of animal intelligence, more especially in connexion with the grey nervous matter which we have traced up from its earlier ganglionic development to the brain of man. "There is an intimate relation (says Solly, page 57,) between the bulk of cineritious neurine, in which each individual nerve of sense terminates, and the perfection of the organ of sense from which that nerve arises."

"The further we advance, indeed, we meet with fresh proofs that the brain, even of the highest order of animals, is no more than a series of ganglia."*

And that, up to a certain point, there is no traceable difference but only in degree between the arrangement, composition, and functions of the brains of the higher class of animals and man. As in him, so in them, the nerves all proceed directly or indirectly to or from the brain,—in him as them, the ganglia receive mere nerves of sensation, whilst all the nerves of sense proceed from the brain;—in them as in him the intelligence is proportionate to the frontal development, and amount of cineritious matter.

Before quitting this part of the subject, it may be necessary to prove that the brain is really and truly the organ of the mind, that is, the peculiar and sole portion of the body which manifests it. As regards the higher class of animals, this is easily done. All the nerves proceed directly or indirectly, as already stated, to or from the brain. If we divide a nerve in any part of the body, all the functions of that nerve will be lost in the part which is cut off from the cerebral connexion, whilst that portion which is still in communication with the brain, retains its power more or less modified by the injury. Thus, if the optic nerve be divided, no image can be received by the retina, or transmitted to the brain. Blindness is a necessary consequence; but if a man's leg be cut off, the nerves of ordinary sensation retain their functions in the stump, often in an aggravated degree, and years after, from long habits of association, pains will be referred to the toes, which are really existing in the thigh. Moreover, every other portion of the body may be mutilated and destroyed, without affecting the manifestations of mind; "but there are no cases on record in which the mental faculties have remained undisturbed, when the disorganization has extended *to both sides of the brain*."†

So far then our theory is established.

1st.—The brain is the organ of the mind.

2nd.—As brain, so mind.

Young or old, healthy or diseased, powerful or feeble, as the brain is, so is all we know of mind, and this can be predicated of no other part of

* Soemmering, p. 65. † Solly on the Brain, p. 349.

the body. But here a question arises which affects the matter seriously. Is consciousness truly the distinguishing characteristic of the animal kingdom, and is it a mental manifestation? How then do animals manifest it who have no brains?

It has been already stated, that the human brain is nothing more than a series of ganglia—and, it is equally true, that the cineritious portion of these ganglia manifests perception and will. Now, down to the caterpillar, we trace not only ganglia, but cineritious neurine. Properly speaking, there is no brain; the single ganglia in the head of the insect performs the function. Lower still in the polypus, whose every portion may be cut away, forming so many new animals, and containing so many centres of life and consciousness, even though the microscope should fail to detect neurine, or even a ganglion, we have the authority of every living thing above it, for declaring that there must be ganglia, though we cannot detect them; there must be neurine though we fail to find it; because ganglia alone receive sensations, and neurine alone perceives them. We are carried, therefore, from the known to the unknown, by a large and unerring analogy, which becomes almost a syllogism, and bears about it marks of mathematical precision.

Thus the most certain, never-wanting attribute, of all animals whose nature we can examine, is consciousness.

The universal concomitant of consciousness is neurine.

Both the above can be traced or inferred to the lowest link of animal life; and

Neither of them can be traced to the vegetable.

We have now to inquire if the mental manifestations, exhibited by other animals and man, differ in kind, or only in degree.

Consciousness has been already named as the primary element of mind; and the term is used as synonymous with perception. All animals perceive or feel, most of them see, many of them hear, taste, and smell. These are intellectual operations, and man exhibits no superiority over the dog or the monkey in this respect; nay, in civilized life he falls far short of their perfect sense. Animals, too, especially of the higher class, become as accurately acquainted with the physical properties of matter as man himself. They perceive and appreciate form, size, weight, and colour with the nicest judgment. Their sense of smell is far more acute than ours, and their hearing as superior. They have musical ears—are conscious of the lapse of time—can construct and count,* though to a very limited extent. It is certain that a dog will fight one dog, and run away from two; he, therefore, knows that two is more than one, and this is nearly equal to some savages who can only count four; whilst the Bidders amongst us can almost multiply the stars of heaven by the sand on the sea-shore, and tell us the result.

Among the perceptive powers, then, those which cognize number, time, and perhaps order, seem to be those generally given more largely to man than to the brute; whilst those which perceive the *physical* properties of bodies, are frequently more exquisitely developed in animals than man. Ulysses' dog alone detected him, whose long wanderings had made

* This was doubted by Spurzheim, and affirmed by George Le Roy. He states that a magpie having stolen some game, it was determined to shoot it. A man hid himself in a hut near its nest for this purpose. The bird flew away when he entered, nor would return. The next day two men entered, and one came out. Mag was not to be cheated, she waited till the second left also. Three went in and two came out with the same result. Four then entered, and three came away. The bird went back and was shot. So magpies, says George Le Roy, can count three but not four!

him equally an alien to the memories, as to the affections, of his courtiers and friends.

By the action of the perceptive powers all knowledge comes into the mind; and this knowledge is directly proportionate to the size and quality of the nerves, ganglia, or brain, which receive the transmitted impressions. Not only will blindness or deafness ensue, if injury be done to the eye or ear, but any damage to that portion of the brain receiving a nerve of sense, produces a corresponding result in the perception; thus deafness may arise from pressure on the brain, and blindness from a tumour within it.

Thus, too, the eye may be perfect, the ear well-formed, the nostril, tongue, and skin in a normal condition, and yet, from malformation of the brain, not only shall the higher class of faculties be wanting, but the perceptions degraded below the lowest animal that crawls or swims.

"William Redfern, who resided about ten years since at Stone, near Kidderminster, aged fourteen years, was a striking instance of this. His eyes were prominent, bright, and apparently perfect; all the external organs of sense, as far as they could be examined, seemed equally well-formed. He exhibited no other mental powers than the following:—'He cries out when hurt; and, what they believe to have been a smile, has occasionally been seen upon his countenance. He eats voraciously of some things, reluctantly of others, and turns round more quickly at the sound of William (his name) than at any other; but beyond this he has been never known to differ from a vegetable. Consciousness is present, and slight traces of memory; but judgment, understanding, and almost volition are nearly wanting.'

"His touch is perfect, but he has never been known to take hold of anything in his life; his hearing is perfect, but, except as regards his own name, they cannot discover that he distinguishes one sound from another.

"His sight is sound, but he knows not his own mother, or one object from another.

"From long disuse his fingers are bent upon his hands, and his legs are permanently crossed. As they place him, so he remains, never moving hand, foot, or head; an occasional whine or motion of the eyes being the only proofs he ever gives of anything approaching to volition."

As far as the perceptions are concerned, then, we have abundant evidence that animals equal man in most, and surpass him in some. That they have memory is equally clear, but this obliges an inquiry into the nature of this supposed primary elementary faculty; for even Reid so considers it.

"In the gradual progress of man, from infancy to maturity, there is a certain order in which his faculties are unfolded." "The external senses first appear—memory soon follows." "Memory must have an object—it must be past."* But enough of quotations, and, in spite of Chalmers, let us look into the workings of our own minds for evidence of their nature. To see is one thing, to look another; to hear is one thing, to listen another; to look, or listen long, attentively, and with highly organized eyes, ears, and brains, is a third; and in proportion to the intensity of our attention, and perfection of the organs, especially of those in the brain, is the power possessed of recalling the impression, of reviving the image, of reproducing the idea which has been received. All the physical properties of bodies can thus be renewed and recombined, long-buried thoughts restored, and words and acts repeated. We can re-quote and re-argue the same ideas and the same words we employed, it may be, years ago; or, shutting our eyes, we can fling ourselves abroad into the world we have

* Reid's Essays, iii. p. 158.

seen, and trace again, in hues as bright and forms as bold as when first we saw them with our bodily organ, the gorgeous temple, or the heaving sea—the mighty mountain, or the battle-plain. But each man knows that his memory is not one, nor his memories equal. The mind is one, but it has many agents. The eye of one man may be more perfect—the ear of another. One may perceive colours, another forms, more clearly. Music may be the attribute of one, mechanics of another. Our faculties differ in power, so do our memories; and it will be found, that, so far from memory being an elementary power of the mind, it is nothing more nor less than *the highest function of an intellectual faculty;* and this at once explains the vast difference observable, not only in various individuals, but even in ourselves, at one or other periods of our lives. 'Tis the last to come, the first to go—most readily affected by illness or fatigue—and requiring the greatest labour to strengthen and retain where the perceptions are feeble, but clear and ready as the perceptions themselves, where they are strong.

Although foreign to our present subject, it may be mentioned that feelings have no memories. We can remember that we relished the feast, or enjoyed the dance; we can enumerate the viands, recal the groups, but can no more reproduce the feeling than we can the objects that created them. Similar feelings may be renewed by similar objects, but the same feelings are gone for ever; we can remember that we *were* happy or sad, and we can even feel sad or happy that we were so. Be this, however, as it may, it is evident that animals differ in nowise from man in the exhibition of perceptive memories, except that herein many of them excel him.

By perceptions animals gain knowledge. The various items of which this knowledge is made up constitute so many ideas, and in dealing with those ideas, we reason. "In reason," says Locke, "there are four degrees; first, the discovering and finding out proofs; second, regularly and methodically arranging them; third, perceiving their connexion; fourth, making a right conclusion;" again: "Reason is that faculty whereby man is supposed to be distinguished from beasts, and wherein it is evident he much surpasses them." Space is wanting to analyze these definitions, but it will be observed, that Locke doubts the supposed distinction between man and beasts, and only asserts positively that our reason greatly surpasses theirs.

What do we when we reason? We take any two or more of our ideas, thoughts, or feelings, and compare them; we cannot compare an idea with itself, but only with something else. For instance, I may compare this book with other books, this page with others, or this line, word, syllable, or paper, with other lines, &c., but I cannot compare any of these with themselves, only with others. And in comparing them or any other things, or ideas of things, I endeavour to find out wherein they differ, wherein they agree, and why they differ, why they agree? These three faculties, then, the one which traces differences, called by phrenologists the faculty of comparing, but which should be named, the power of analyzing; secondly, the power of tracing resemblances, improperly named wit; and thirdly, the power of connecting causes and consequences together, constitute, as far as I can judge, the whole of our reasoning faculties, and they are employed in every relation of mind; in knowing a tree when we see it, or in distinguishing a tree from a stone, a house from a man, earth from water, or straw from grass, truth from falsehood; by these three powers the poor idiot distinguishes his food from fire, takes one and repudiates the other; by them he goes to his bed, or rises, or walks, or warms himself; by them, and only them, Newton followed the planets in their orbits, tracked the comet in its wildest flight, showed that charcoal and diamond were the same, and that water ought to burn.

And does man then only reason? Is instinct the brutes' only prerogative? What is instinct? It has been well defined, as "*knowledge before experience.*" It is the desire to do and the power to do, as the progenitors have done before in all time, without varying, without change, the like thing in the like manner. The bird builds its nest each after its kind, of the same shape, and if it can get them, of the same materials, as it has been built in all time by those who have preceded it; and it does this without instruction, without experience. Hatch a blackbird, a swallow, and a thrush, in cotton in a cage; let them free when grown, and in the coming spring the blackbird will line her nest with hay, the thrush with clay, and the swallow will carry mud from pond or river to build up the walls of her house in your chimney, as her ancestors of yore have ever done.

The spider hangs her nest upon the branches, or spreads her gossamer over the fields; the squirrel, unknowing that winter is coming, or that nuts will disappear, garners up a store in some hollow beech tree, and sleeps secure of her bonded corn when winter shall have past away, and nor fruit nor grain be found.

It is the very essence of instinct to be unteachable and unimprovable; so perfect has it ever been and is, in all its works, that Sir Isaac Newton always believed it to be the spirit of God directing the wings and beaks of birds, antennæ of bees, and spider's limbs; for that no second agent could be so infallible.

But the essence of reason is, that it is knowledge after experience; it is built up by a comparison of facts, and by tracing their relations and results; it grows with our growth, and strengthens with our strength. It not only can be taught, but exists only by teaching; it is fallible, whilst instinct never errs; it is progressive, instinct immutable; reason is indefinite, instinct limited and defined; each man acquires his own results, whilst instinct is given at birth. In proportion as we ascend the scale, we find instinct becoming less and less apparent. Whilst the ant, the spider, the bird, and the bee are beautiful models of it, the elephant, the dog, and the monkey exhibit scarcely a trace. Man none at all.

What intelligence have they then, if wanting in reason, and deficient in instinct? * Instead of being the most, they should be the least sagacious of all animals; but if the above views are correct, they have reason approximating closely in amount, and identical in quality with that which has so long been thought to belong exclusively to man; and so has the bee, and the fish, and the serpent, and all things having brains that are to be found "in the heavens above, or the earth beneath, or the waters under the earth." Instinct tells them what to do, reason how to do it.

The bee builds her cells of the hexagonal shape, flies *from* some flowers and to others, swarms and makes honey and wax, instinctively; but reason shows it the way. It dashes not itself against houses and trees; it compares one hive with another, enters the right, avoids the wrong; knows a drone from a working bee, cherishes the one, and destroys the

* One of the most striking instances of instinct occurred in a Newfoundland dog belonging to the author. She was going down to St. Albans with her master, when, suddenly she sniffed the air, started back, and evidenced every symptom of intense fear. By encouragement she would advance a few paces, then dash back again, and cower at his heels. On looking for the cause of her alarm, Wombwell's caravan was observed about three hundred yards in advance!! The animals made no noise, or if they had, the dog was not a twelvemonth old, and had never seen or smelt a wild beast in her life!

other. Instinct leads the salmon from the deep sea through unknown ways to the topmost rill of the running stream; instinct compels it to burrow in the gravel there to deposit its spawn, but reason guides it in its course, and directs its operations.

Cannot the elephant be taught? does he not compare, analyze, trace similitudes, calculate results? Then how is it he learns to fire off pistols, unbolt doors, hold out his trunk for halfpence, cry "cake!" and exchange his money for the dainty, like any school-boy? Will not the goldfinch learn to draw up water in his cage; and the dog to go where he is bidden, to fetch and carry, guard the flock, or bring it home as his master may direct? The very pig may be taught to pick up one card in preference to another, by comparing the tones of his master's voice, and associating them in his memory with the object to be accomplished.

But we must have, forsooth, evidence of remote inferences, abstract comparisons, before we grant reasoning powers to the brutes. We are not content with those powers of comparison which "guard his master from a post," and enable the horse to enter the stable-door when it is open, and refuse the attempt when it is shut! We deny the name of reason to the mental operations, which make the lamb fly from the dog that has bitten it, to the mother that protects it, and enables that mother to single its own out of ten thousand.

Huber relates that bees always commence building their combs at the top. They cannot build upwards, but always down.

Now, a high wind shook one of his hives, and detached a comb. The bees immediately wedged it firmly in between the others, lest it should damage them; and they did more—they immediately set to work, *and strengthened the attachments* of all the other combs, to protect them from a similar accident!

Kirby and Spence relate how a sparrow robbed a swallow (quere martin) of her nest, and how the swallows consulted together, went in a body to the pond, flew back each with some mud in its mouth, built up the entrance to the nest, and made him a prisoner, as the monks of old did their victims, not only for life, but unto death. *He was walled in, and starved!* An animal, then, can neither walk, swim, or fly, feed or sleep, without exercising the whole of the reasoning powers attributable to man; and his reason differs from ours, not in kind, but only in degree: it constitutes our pre-eminence, but not our prerogative, for the poor idiot and the child exhibit far less of it than the beast of the field or bird of the air. Now, if this be so, why are animals stationary? Why do they not progress as man progresses? Why do they not construct machines to shorten labour? Why do they not help each other? Why cannot they hand down their knowledge to their offspring? It is not for want of hands; for the monkey has them. It is not for want of memory;—Ulysses' dog was but a sample of his kind. Nor from lack of judgment; for they know, therefore they have judged. And when a horse or dog obeys the voice, to stop or go, stand up or lie down, or do the thousand things they learn to do, they evidence by their actions that they understand the tone, the manner, and the object. They crouch or come, as the harsh or harmonious in sound or gesture meet or eye or ear; and *understand* the meaning of the uplifted hand and whip as well as the veriest urchin, who is conscious he deserves it, and sees it coming. The very pig distinguishes the difference between the sound of the pail that brings his meal, and any other in which he has no interest, be it wheelbarrow or cart: how he cries in his joy, as he hears the bin-lid fall, and footsteps going thence to him. It is because reason, and memory, and judgment, and will, have been assumed to be elementary powers of the mind, instead of terms describing intellectual operations and results, that brutes have been thought to want them; and it is because animals have instinct, and man has not, that it has been supposed amply sufficient to account for all their doings; although every thinker on the subject, and almost every writer, from Plato to my Lord Brougham, have felt that, if animals have not reason, they have something so very like it, that it is hard to draw the line. I hope to have shown that the operations of instinct are limited and well-defined, and that reason does not constitute the *intellectual difference between other animals and man!* Why, then, again, do we find no other class of animals than man capable of improvement? The answer is obvious. *They have no articulate speech!* They have intellect, feelings, and tongues; they have the power by inarticulate sounds of making their feelings known, but not their knowledge.

The sentry crow flies off, and screams when fox or gun comes near, and all fly off and scream: it is frightened, so are they, but it alone knows why. It cannot tell to them the cause of its alarm, neither can dog or horse impart his cunning. Each begins life for himself, and by himself must he gain his own knowledge: however great this may be—though he shall learn to fetch and carry, obey commands, fire guns, mimic death, and do the thousand imitations of humanity exhibited by beast and bird, even to the senseless imitation of our speech—yet have they no means of handing down to their offspring the smallest information on any point whatever; and so, although the individual animals grow "old and wise," the class remains as it was created—it "dies and makes no sign." Man, too, has inarticulate language as even have the beasts, which, when his heart is bursting with sorrow or with joy too deep for words, supplies their place, and, by reducing him to the level of a brute, emulates its truthfulness. The cry of terror or delight can neither be simulated nor deceive; and "childhood's moan o'er a mother's grave" is as beautiful and true as the dog's wailing howl for its slain companion. But man has more than this—he has *articulate speech!* He alone can associate sound with sense,—change the fleeting tones of his voice into arbitrary and conventional signs, —paint images in the air, as palpable to the ear as solid matter to the eye, —change sound into substance,—"give to airy nothing a local habitation and a name," and fix imperishably in stone or brass not only his feelings but his experience—not only what he felt, but what he did; and why he did it. He alone can communicate his knowledge, and that not only to those about him, but to distant lands and times: so that each child begins life upon his father's shoulders. The experience of ages constitutes the base of his own temple, and that of his child's children down to the remotest generation. Each after each will be spared *his* trouble; all they have to do will be to add to the mighty Babel of human knowledge aught they may discover of the new and the true; and, however small the contribution, the man who adds but one brick to the building may exclaim, with Horace, "Exegi monumentum ære perennius!" Intellectually, therefore, *articulate language*, and that alone, constitutes the distinction and the supremacy which separate man from every living animal but himself; and if it be objected that some are dumb even from birth, I answer, that some lose their legs and some their arms,—some are deaf, and some are blind; and yet, legs, and arms, and eyes, and ears, essentially belong to the genus man; and the accidental privation of either affects not his description.

Animals have all his perceptive powers,—all his reflective. In the feeble development of these latter, however, is to be found the *degree* of inferiority which belongs to them. They can analyze and trace analogies, —they can readily connect consequence and cause,—but all these intellectual processes are limited to their own personal wants and experience, bounded by their own mental horizon. They cannot reason upon abstractions,—not because they want reason, but because they have no *abstract ideas;* and why they have none will be determined when we come to investigate, should an opportunity be allowed, *the moral difference between other animals and man.*

